# Infant Pneumococcal Carriage in Belgium Not Affected by COVID-19 Containment Measures

**DOI:** 10.3389/fcimb.2021.825427

**Published:** 2022-01-17

**Authors:** Laura Willen, Esra Ekinci, Lize Cuypers, Heidi Theeten, Stefanie Desmet

**Affiliations:** ^1^ ﻿Centre for the Evaluation of Vaccination, Vaccine and Infectious Disease Institute, University of Antwerp, Wilrijk, Belgium; ^2^ Department of Laboratory Medicine, National Reference Centre for Pneumococci, University Hospitals Leuven, Leuven, Belgium; ^3^ ﻿Laboratory of Clinical Bacteriology and Mycology, Department of Microbiology, Immunology and Transplantation, Katholieke Universiteit Leuven, Leuven, Belgium

**Keywords:** *Streptococcus pneumoniae*, nasopharyngeal carriage, invasive pneumococcal disease, COVID-19, containment measures

## Abstract

*Streptococcus pneumoniae* is an important and frequently carried respiratory pathogen that has the potential to cause serious invasive diseases, such as pneumonia, meningitis, and sepsis. Young children and older adults are among the most vulnerable to developing serious disease. With the arrival of the COVID-19 pandemic and the concomitant restrictive measures, invasive disease cases caused by respiratory bacterial species, including pneumococci, decreased substantially. Notably, the stringency of the containment measures as well as the visible reduction in the movement of people appeared to coincide with the drop in invasive disease cases. One could argue that wearing protective masks and adhering to social distancing guidelines to halt the spread of the SARS-CoV-2 virus, also led to a reduction in the person-to-person transmission of respiratory bacterial species. Although plausible, this conjecture is challenged by novel data obtained from our nasopharyngeal carriage study which is performed yearly in healthy daycare center attending children. A sustained and high pneumococcal carriage rate was observed amid periods of stringent restrictive measures. This finding prompts us to revisit the connection between nasopharyngeal colonization and invasion and invites us to look closer at the nasopharyngeal microbiome as a whole.

## Introduction

*Streptococcus pneumoniae* is part of the commensal flora of the upper respiratory tract and colonizes, together with several other bacterial species, the nasopharyngeal niche. Even though its residency in the nasopharynx typically goes unnoticed, when natural immunological barriers are crossed, respiratory or even systemic disease ensues. Pneumococcal colonization is, thus, imperative for disease to occur and is responsible for the horizontal spread of the different pneumococcal serotypes within the community. Close contact between individuals, and therefore crowded places, adds to the spread of pneumococci (Bogaert et al., 2004). Young children are particularly known to frequently possess pneumococci, even more so when attending daycare centers, and especially so during the drier and colder months when its spread is more likely to coincide with respiratory viral infections (Numminen et al., 2015). Pneumococcal conjugate vaccines (PCV) were developed to prevent (invasive) pneumococcal disease caused by the, at the time, most invasive serotypes. In countries with a high vaccine uptake, this has led to decreases in both carriage and cases of invasive pneumococcal disease (IPD) caused by the vaccine serotypes (Cohen et al., 2015; Vissers et al., 2018; Wouters et al., 2020; National Reference Centre for invasive S. pneumoniae, 2021). However, the nasopharyngeal niche is a complex and highly dynamic environment with high turnovers of colonizing species and serotypes. Vaccinating against the most invasive pneumococci, thus, results in their replacement by other serotypes in the nasopharyngeal niche (Bogaert et al., 2004). Evidently, monitoring the distribution of the different pneumococcal serotypes within populations is essential to inform vaccination policy and direct future vaccine development. As a result, many countries worldwide have performed nasopharyngeal carriage studies, some of which are still ongoing (Cohen et al., 2015; Vissers et al., 2018; Wagner et al., 2019; Wouters et al., 2020). In Belgium, our group has been monitoring asymptomatic pneumococcal carriage in daycare attending children (aged between 6-30 months old) every winter since 2016, with carriage rates fluctuating between 68 and 74% (Wouters et al., 2020) (unpublished data).

In response to the outbreak of the COVID-19 pandemic in early 2020, countries worldwide laid down a range of restrictive measures to contain the further spread of the SARS-CoV-2 virus. This has led to the discontinuation of several pneumococcal monitoring studies in many countries. Also, in Belgium, this appeared challenging. However, with great effort comes great reward: despite the many restrictions imposed on the Belgian people, monitoring nasopharyngeal carriage was not interrupted and the target number of recruited children was obtained. More importantly, high sustained carriage rates were measured during this period. The importance of our findings was revealed when the ﻿Invasive Respiratory Infection Surveillance (IRIS) Initiative published international surveillance data of invasive bacterial diseases in *The Lancet Digital Health* (Brueggemann et al., 2021). They confirmed the much-anticipated impact of country-wide lockdowns on the incidence of invasive disease caused by the three most common respiratory pathogens *S. pneumoniae*, *Haemophilus influenzae*, and *Neisseria meningitidis*. Significant and sustained reductions were observed, based on surveillance data from ﻿37 laboratories from 26 countries worldwide, that appeared to coincide with the stringency of COVID-19 containment measures ﻿(measured using the Oxford COVID-19 Government Response Tracker) and ﻿with changes in the movement of people (measured using Google COVID-19 Community Mobility Reports).

## The Belgian Situation: Stringent Measures, but High Pneumococcal Carriage Rates

**Figure 1:**
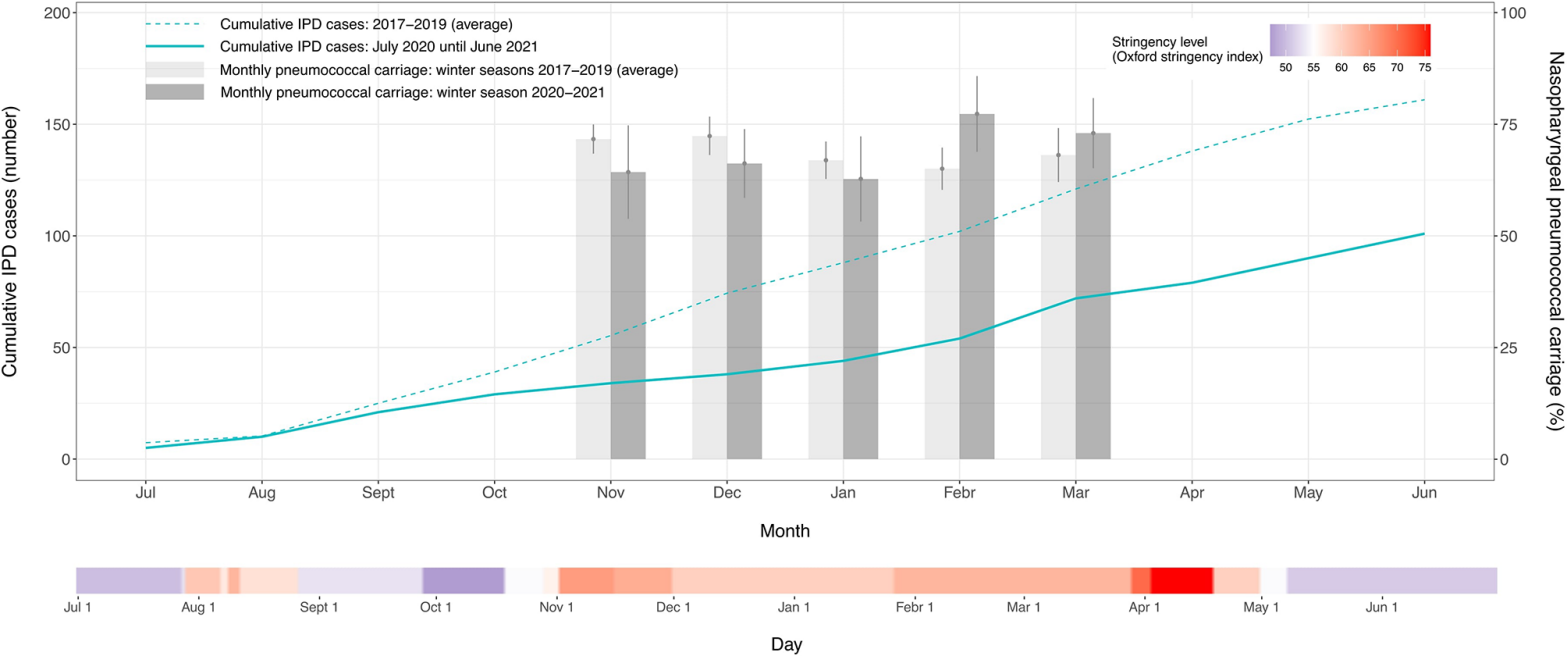
*Streptococcus pneumoniae* carriage rates and invasive pneumococcal disease cases in young children (aged <3 years) obtained from July 2020 until June 2021 and compared to the previous three epidemiologic years. The cumulative number of invasive pneumococcal disease cases and the average pneumococcal carriage prevalence are respectively shown as a monthly total and a monthly percent proportion. The vertical lines on the bars represent the 95% confidence intervals of the monthly percent carriage proportions. The time point indicated on the x-axis represents the middle of each month. A second continuous axis shows the daily level of stringency of the COVID-19 containment measures implemented in Belgium from July 2020 until June 2021. The stringency index is based on the Oxford COVID-19 Government Response Tracker and is shown on a gradient color scale going from 0 to 100, with red values indicating that higher stringency measures were operating.

In Belgium, the government announced a lockdown on March 18, 2020, thereby ordering the closing of shops, and enacting stay-at-home orders and travel restrictions. This resulted in a drastic reduction in movement as indicated by corresponding mobility data (Sciensano, 2021). A gradual relaxation of the containment measures from May 2020 led to a worsening COVID-19 epidemiologic situation and, consequently, new restrictions were gradually implemented from early October 2020 until late April 2021 (Oxford stringency index > 55) (**Figure 1**). Analogous to the observations made by IRIS, also this second period of restrictive measures in Belgium – corresponding to the winter of the epidemiologic year 2020-2021 – was characterized by a reduction in IPD cases of 42% in young Belgian children (aged <3 years) when compared to previous non-COVID epidemiologic years (2017-2019). As a result, the pronounced seasonality typical for IPD did not manifest itself in the winter of 2020-2021 (**Figure 1**).

These reductions in disease incidence could be explained by an interruption in person-to-person bacterial respiratory transmission (Brueggemann et al., 2021). While plausible, high pneumococcal carriage rates recorded during the same period seem to suggest otherwise. In the winter of 2020-2021, we did not detect a significant change in overall *S. pneumoniae* carriage proportion (67.43%) compared to the average of the previous three years (69.07%) (*X* ^2^ = 0.56, *P*=0.46) (**Figure 1**). The observed discordance between IPD cases and carriage prevalence in the same age group may, thus, point to a more complex situation and possible interspecies interactions in the nasopharyngeal microbiome.

Even though bacterial colonization is a necessary precursor for invasive disease, it does not imply the occurrence of disease per se, nor do we understand all the interspecies interactions or host factors that should coalesce for the bacteria to become invasive. Altered transmission of other species inhabiting the nasopharyngeal microbiome could also have contributed to the reduced IPD cases (Bogaert et al., 2004). Viral respiratory infections are known to be associated with IPDs (Weiser et al., 2018; Berry et al., 2020). Therefore, a more fitting hypothesis, and previously raised by Smith et al. (Smith and Opatowski, 2021), is that the COVID-19 containment measures reduced the overall respiratory viral circulation, which could have eventually impacted the incidence of IPD in children. It is thought that the inflammatory conditions in the upper respiratory tract following a respiratory viral infection both favor the presence and transmission of *S. pneumoniae*, and increase its likelihood of penetrating host tissues (Weiser et al., 2018). However, recent work revealed that while influenza-like illnesses (ILIs) indeed increase the pneumococcal invasion risk, it does not increase their transmission or acquisition risk (Domenech de Cellès et al., 2019). Consequently, interventions targeting ILIs were predicted not to have a marked indirect effect on pneumococcal carriage – a prediction confirmed by the sustained high carriage rates observed in our study. Importantly, we did not assess pneumococcal carriage density, a precursor of IPD, and known to be elevated in individuals with ILIs (Brotons et al., 2017; Miellet et al., 2021). Possibly also SARS-CoV-2 co-infections could have impacted pneumococcal carriage rates (Howard, 2021); however, in our study, none of the tested children (746/907, 82%) was positive for SARS-CoV-2 (unpublished data).

## Scrutinizing the Relationship Between Pneumococcal Carriage, Transmission, and Invasion: What Else to Consider?

It can, however, not be ruled out that along with a decrease in the co-circulating viral population, also a reduced frequency of within- and between-age contacts in the overall pediatric population has impacted IPD development (Domenech de Cellès et al., 2019). Both pneumococci and respiratory viruses, including SARS-CoV-2, can be transmitted from person-to-person *via* respiratory secretions through direct physical contact or directly through the air *via* large respiratory droplets (Weiser et al., 2018; Leung, 2021). Airborne transmission, on the other hand, is likely more important for the transmission of respiratory viruses/SARS-CoV-2 than for pneumococci. However, it should not be dismissed as a possible transmission route for the latter: a study on ferrets living in cages three meters apart from each other did observe transmission of pneumococci between them (McCullers et al., 2010). Lastly, just like respiratory viruses and SARS-CoV-2, pneumococci can also be transmitted indirectly *via* contact with contaminated surfaces or objects (fomites), with the duration of their survival in the environment depending on the situation, going from several hours in nutrient-poor conditions to a couple of days in e.g. saliva or biofilms (Marks et al., 2014; Hamaguchi et al., 2017; Marzoli et al., 2021; Morimura et al., 2021). Notably, in daycare settings, pneumococci were seen to survive for hours and could be cultured from environmental surfaces, including toys (Marks et al., 2014). Given the parallel routes of transmission of both pneumococci and respiratory viruses, restrictions that were primarily aimed at reducing transmission of SARS-CoV-2 might, thus, also have affected pneumococcal transmission in the overall pediatric population. Importantly, during the country’s first lockdown (March-May 2020), Belgian daycare centers remained open but were only occupied for 50%. Since we do not have carriage data of this period or its aftermath, we cannot tell if transmission between children declined following reduced contacts. On the other hand, in the winter of 2020-2021, the number of children attending daycare was in line with previous years, and essentially little restrictions were imposed, likely adding to the sustained high carriage rates. Adding to this, and if we want to understand the epidemiology of the pneumococcus entirely, it is paramount to also define (yearly) pneumococcal carriage rates in Belgian children who do not attend daycare centers [i.e. 40% of the Belgian children, (OECD, 2021)].

Of course, the carriage prevalence reported here only depicts the situation in Belgian daycares, a high transmission setting in which changes in the overall pneumococcal carriage might be hard to discern. Given the marked differences in the capacity of specific *S. pneumoniae* serotypes and clones to cause invasive disease (Brueggemann et al., 2003), individual serotype carriage proportions should be disentangled to better understand how such an overall high carriage rate relates to the observed drop in IPD cases. In Belgium specifically, serotype distributions might have shifted given the country’s switch in PCV recommendation in September 2019, one year prior to our study. That year, PCV13 replaced PCV10, extending children’s protection to an additional three serotypes. Of note, the impact of an earlier switch in the Belgian PCV program in 2016 – from PCV13 to PCV10 – was only detectable two years after its implementation (Wouters et al., 2020). Likely a similar interval is needed to detect the impact of the second switch. More so, IPD surveillance in Belgium indicated that in 2020 the same proportion of serotype 19A strains (43.8%) were responsible for IPD in children <2 years of age compared to that in 2019 (39.4%) (National Reference Centre for invasive S. pneumoniae, 2021). Nonetheless, shifts in the distribution of circulating serotypes and in the multilocus sequence types of pneumococcal serotype 19A following the 2019-switch in PCV recommendation are being investigated, and could be instructive for even better understanding how overall high carriage rates relate to the observed drop in IPD cases. A relation that could, in fact, be an amalgam of all players discussed here, including (but not limited to) respiratory viruses, serotype replacements, and reduced contacts.

## Conclusion

The sustained high asymptomatic carriage patterns observed in our study reveal that IPD incidence does not solely depend on pneumococcal carriage rates, and that likely many more factors are at play. Our finding, thus, puts the relationship between high pneumococcal carriage rate and invasive disease into perspective and sheds a light on the potential role of the co-circulating viral populations when it comes to IPD incidence in young children. Evidently, we should not narrow down on a single component cause, nor should we devalue the importance of person-to-person bacterial respiratory transmission. When unraveling the epidemiology of the pneumococcus many factors need to be considered. We believe that our results and insights presented here will be able to assist further pneumococcal research. Especially so since results like these are scarce, considering most countries were not able to continue monitoring nasopharyngeal carriage during the COVID-19 pandemic.

## Data Availability Statement

The raw data supporting the conclusions of this article will be made available by the authors, without undue reservation.

## Ethics Statement

The studies involving human participants were reviewed and approved by Comité voor Medische Ethiek, University Hospital Antwerp, Edegem (Belgium). Written informed consent to participate in this study was provided by the participants’ legal guardian/next of kin.

## Author Contributions

HT acquired funding, conceptualized, and supervised the study. LW, EE, LC, and SD analyzed and verified the underlying data. LW wrote the first draft of the manuscript and visualized the data. All authors reviewed and edited the manuscript, had full access to all the data in the study and had final responsibility for the decision to submit for publication.

## Funding

UZ Leuven, as the national reference center for pneumococci, is supported by Sciensano, which is gratefully acknowledged. SD and HT received an investigator-initiated research grant from Pfizer. The study is also supported by a research grant from Research Foundation Flanders (FWO Research Grant 1150017N, Antigoon ID 33341). The funders had no role in the study design, the collection, analysis, and interpretation of data, the writing of the report, or in the decision to publish.

## Conflict of Interest

SD and HT received an investigator-initiated research grant from Pfizer.

The remaining authors declare that the research was conducted in the absence of any commercial or financial relationships that could be construed as a potential conflict of interest.

## Publisher’s Note

All claims expressed in this article are solely those of the authors and do not necessarily represent those of their affiliated organizations, or those of the publisher, the editors and the reviewers. Any product that may be evaluated in this article, or claim that may be made by its manufacturer, is not guaranteed or endorsed by the publisher.
